# Preparation of Nano-Sized C-S-H and Its Acceleration Mechanism on Portland Cement Hydration at Different Temperatures

**DOI:** 10.3390/ma16093484

**Published:** 2023-04-30

**Authors:** Yanfeng Fang, Limin Zhang, Li Li, Mingyu Zhao, Qing Wang, Yong Mei

**Affiliations:** 1 School of Materials Science and Engineering, Shenyang Jianzhu University, Shenyang 110168, China; fangyf@sjzu.edu.cn (Y.F.); zlm18234297506@163.com (L.Z.); zmyzhaomingyu@sjzu.edu.cn (M.Z.); 2 College of Water Resources and Architectural Engineering, Northwest A&F University, Xianyang 712100, China; drlili@nwafu.edu.cn; 3 Institute of Defense Engineering, Academy of National Defense Engineering, Academy of Military Sciences, Beijing 100036, China; meiyong1990@126.com

**Keywords:** nano C-S-H suspension, size distribution, hydration, early strength, morphology

## Abstract

Nano-sized C-S-H, a promising early strength agent, can accelerate the hydration rate of Portland cement and increase the early compressive strength of cement-based composites effectively. Nano-sized C-S-H suspensions with different contents of effective constituent and size distributions were prepared by a convenient coprecipitation method and the microstructures were analyzed by Zeta potential, XRD and FT-IR. The exothermic heat, early mechanical properties, hydration degree and hydration products of cement with/without nano-sized C-S-H cured at different temperatures were studied by hydration exothermic, XRD, SEM and TG analysis. Nano-sized C-S-H with semi-crystalline structures was prepared, and the size of the nano-sized C-S-H seeds showed an obvious increase with an increase in theoretical concentration, and slight precipitation in the suspension was observed when the theoretical concentration was 2%. The XRD, TG and SEM analyses showed that nano-sized C-S-H expedites the reaction of C_3_S in the first 24 h; therefore, the hydration induction period is obviously shortened. The 8 h, 16 h and 24 h compressive strength of mortars containing nano-sized C-S-H increased by 176.0%, 145.6% and 43.9%, respectively, compared with the reference mortar. The enhancement effects of nano-sized C-S-H at 10 °C were lower than that at 20 °C.

## 1. Introduction

The engineering community has been constantly developing new materials and new technologies to satisfy the early strength requirements of industrial development [1,2]. For instance, low-temperature construction and the production of concrete prefabricated components often employ steam curing or early strength components to increase the early strength of concrete [3]. Nevertheless, steam curing showed unfavorable effects on the internal pore structure of concrete [4,5,6], while the traditional early strength agents are mainly inorganic, organic and composite early strength agents, which may lead to the long-term occurrence of alkali aggregate reaction [7]. At the same time, it further accelerates the penetration of corrosive ions into the interior to induce the synergistic effects of alkali aggregate reaction, reinforcement corrosion and freeze-thaw damage, thereby causing adverse effects on the durability of concrete [8,9]. The rapid development of nanotechnology and nanomaterials has brought innovative technologies to the traditional cement industry, and also provided new ideas for the industrial transformation of the traditional cement industry [10]. Research shows that nano-sized materials are a promising candidate for improving the mechanical properties at the early stage [11,12,13]. C-S-H (xCaO·ySiO_2_·zH_2_O) gel is the most dominant composition of hydration products [14,15], and contributes the most to the improvement in the compressive strength of concrete and mortar [16,17]. It is also generally believed that C-S-H is an important component affecting the durability of concrete [18]. Jonh [19] et al. reviewed the synthesis methods of C-S-H and indicated that pozzolanic, sol-gel and precipitation methods were commonly used. The artificially synthesized calcium silicate hydrate shows similar chemical compositions and microstructure to familiar C–S–H formed in a hardened cement-based matrix [20,21]. Nano-sized C-S-H improves the early mechanical properties of cement-based materials by providing nucleation for the growth of C-S-H gel and reducing the nucleation barrier of C–S–H during hydration [22,23,24,25]. Nano-sized C-S-H can also promote the conformation of an appropriate amount of AFt filling the pore structures in the hardened matrix and further improve the cement paste structure [26,27]. At the same time, it will not adversely reduce the development of long-term mechanical properties [20,28,29]. Therefore, it has great application potential in low-temperature construction, maintenance and rescue, production of prefabricated components and other projects.

The synthesis and application of C-S-H seeding for promoting the cement hydration process was reviewed by Cuesta [30], and it was reported that laboratory nano C-S-H seeds and nano C-S-H-based commercial admixtures were widely studied to increase the early compressive strength of cement-based materials. The compressive strength improvements in the early stages depend on the types and dosage of nano C-S-H. The acceptable dosage was in the range of 0.5–2%, and the 1 d compressive strength gain varied within the 9–270% range [19,30]. Nevertheless, the detailed information about the concentration of C-S-H seeds was not reported in many studies, especially in commercial nano C-S-H. At the same time, through market research, we learned that C-S-H seeds are not widely used in the market due to their high cost and high dosage and it is therefore practical to reduce the dosage of nano C-S-H.

The size distributions of nano C-S-H is an important factor affecting the early reinforcement effects [31,32]. Theoretically, there is a negative correlation between the size of nano C-S-H and its nucleation effects; therefore, nano C-S-H with smaller size always results in a higher early strength for a cement-based matrix. Sun et al. [33] synthetized nano C-S-H with the assistance of polycarboxylate superplasticizer (PCE) and showed that the early compressive strength of mortar was increased obviously even with a small dosage of nano C-S-H. Kanchanason et al. [34] synthetized nano C-S-H with different particle size distributions and indicated that the acceleration effect was stronger when the same mass and smaller particle sizes of nano C-S-H were used. However, reports about the effects of concentrations of nano C-S-H on its size distributions are limited, and its effects on accelerating the hydration mechanism and early strength improvement for mortar or concrete is not clear.

In this paper, stable C-S-H suspensions with different concentrations and size distributions were prepared by a convenient co-precipitation method of polymer incorporation, and the stability of suspensions, the microstructure, and the effects of different nano-sized C-S-H suspensions on the early stage hydration performance of cement at 20 °C and 10 °C were investigated.

## 2. Materials and Experiments

### 2.1. Raw Materials

Analytical Ca(NO_3_)_2_·4H_2_O, Na_2_SiO_3_·9H_2_O and NaOH were provided by China National Pharmaceutical Group Co., Ltd. Polycarboxylate dispersant (PCE) was selected as dispersant for the preparation of nano-sized C-S-H suspensions. P·O52.5R cement and IOS sand were selected to prepare pastes and mortars. The chemical compositions of cement determined by X-Ray Fluorescence Spectrum(XRF) are listed in Table 1. Grade I fly ash, ultrafine slag powder, river sand and coarse gravel were used for the preparation of concrete specimens. The apparent densities of ultrafine slag and grade I fly ash were 2625 kg·m^−3^ and 2375 kg·m^−3^, respectively. The specific surface areas of ultrafine slag and grade I fly ash were 904 m^2^/kg and 387 m^2^/kg, respectively. The fineness modulus of river sand was 2.6, and the particle size of coarse gravel was in the 5–25 mm range. The chemical compositions of ultrafine slag powder and grade I fly ash obtained from XRF are presented in Table 2.

### 2.2. Preparation of Nano C-S-H

PCE (solid content of 40%) was used as precipitation template and stabilizer, and Ca(NO_3_)_2_·4H_2_O (38 wt %) and Na_2_SiO_3_·9H_2_O (25 wt %) solutions were used to provide calcium and silicon, respectively. NaOH was dropped to adjust the pH of the obtained suspensions. Nano-sized C-S-H suspension specimens with calcium/silicate molar ratios of 1.2 were prepared by the coprecipitation method. The PCE solution was diluted to 3% with deionized water, and placed at the bottom of a flask. Then, NaOH was dropped to ensure the pH value of the suspension was at 12 ± 0.2. Then, the Ca(NO_3_)_2_·4H_2_O and Na_2_SiO_3_·9H_2_O solutions with different mass fractions were dropped to the PCE solutions (0.14 mL/min) with a high-precision peristaltic pump, while the high-speed shearing machine was stirred at 5000 r/min. Nano-sized C-S-H suspension specimens with theoretical concentrations of 1.0% (S1), 1.5% (S2), 2.0% (S3) and 2.5% (S4) were prepared. The size distributions of nano-sized C-S-H were tested with a zeta potentiometer after two weeks. The Na^+^ and NO_3_^-^ in nano-sized C-S-H were filtrated with deionized water 5 times, dried at 60 °C for about 24 h and ground to the desired particle size for analysis. 

### 2.3. Preparation of Mortar, Paste and Concrete

Mortars containing 5% nano-sized C-S-H suspensions and reference mortar (without nano C-S-H) were designed according to the Chinese standard “Test method of cement mortar (ISO), GB/T 17671-2021” to explore the influence of nano-sized C-S-H on the flexural strength and compressive strength of mortars at early stages (8 h, 16 h, 24 h and 72 h). The w/c ratio was 0.35 for all the mortar specimens. Meanwhile, pastes with/without nano C-S-H suspensions were prepared and cured to specified ages for testing the hydration degree of the cement and the microstructure of the hydration products. For mortar and paste specimens cured for 8 h, 16 h and 24 h, the specimens were demolded until the test age. Specimens for 3 d were demolded after 24 h of curing and thereafter were maintained until 3 d in a standard curing room (98% RH). To compare the acceleration performance of nano-sized C-S-H at different temperatures, two groups of cement mortars and pastes were prepared and cured at 20 ± 2 °C and 10 ± 2 °C, respectively. Concrete containing 5% nano C-S-H suspensions were also cast, and the mix ratios of concrete are presented in Table 3. For each mix proportion, 3 cubic concrete specimens 100 × 100 × 100 mm^3^ in size were cast. All the concrete cubic specimens were demolded after 24 h of curing, and thereafter, specimens were maintained in a concrete room (98% RH, 20 °C) until testing.

### 2.4. Test Methods

The flexural strength and compressive strength of mortars cured to different ages were tested using a universal testing instrument, and the loading capacity of the instrument was 300 kN. For flexural strength, 3 specimens were tested and averaged. For compressive strength, 6 values were averaged. The 1 d, 7 d and 28 d compressive strengths of concrete were tested employing a compressor (loading capacity of 1000 kN). For compressive strength, 3 specimens were tested and an average value was calculated. The paste specimens cured to the specific age were firstly broken and immersed in isopropyl alcohol (C_3_H_8_O) for 24 h, and then the samples were dried at 60 °C for 24 h. The dried and ground powders were stored in airtight plastic bottles until tested.

XRD analyses of synthetic C-S-H powder and ground hydrated cement paste powder were conducted using a D8 Advance X-ray diffractometer. Fourier transform infrared (FT-IR) spectrometer (Equinox 55,from Bruker, Germany) was employed to analyze the structure of the nano C-S-H. Thermal gravimetric analysis of ground cement pastes was conducted using a METTLER TOLEDO TG/DSC1 (from Zurich, Switzerland) system (10 °C/min, N_2_ flow: 50 mL/min) and the fractions of the hydration products in the pastes were examined. Scanning electron microscope(SEM) analysis (Hitachi, S4800, from Tokyo; Japan)was conducted to evaluate the hydration progress qualitatively and the morphology of the content of the hydration products was observed. The heat flow of pastes with/without C-S-H suspensions was analyzed using a TA Instruments TAM Air isothermal calorimeter from New Castle, Delaware.The environmental temperature for testing and the test duration were set at 22 °C and 48 h, respectively.

## 3. Results and Discussion

### 3.1. Stability and Size of Nano-Sized C-S-H Suspension

**Figure 1:**
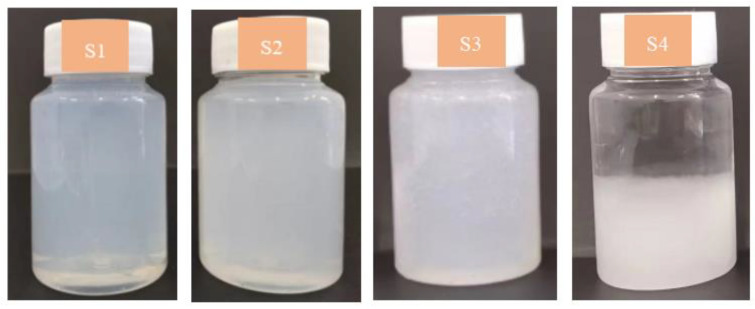
Appearance of synthetic nano C-S-H.

The stability of nano-sized C-S-H suspensions were tested by the visual sedimentation method. The appearance of synthetic specimens standing for two weeks is presented in Figure 1. No layered settlement was observed in the S1 and S2 specimens, indicating that synthetic nano-sized C-S-H suspensions show favorable stability. Nevertheless, slight precipitation in the S3 and S4 suspensions were observed.

**Figure 2:**
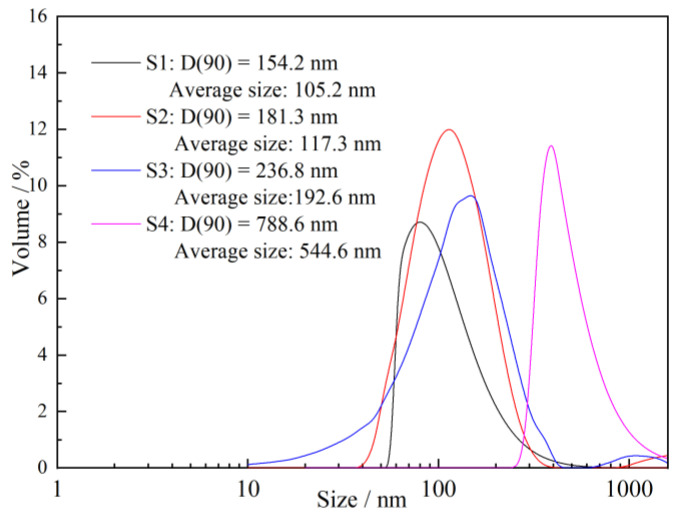
Size distribution of nano-sized C-S-H suspensions.

The size distributions of different nano C-S-H are depicted in Figure 2. The average size of nano C-S-H is positively correlated to the theoretical concentration. The D(90) values of S1 and S2 are 105.2 nm and 117.3 nm, respectively. The average sizes of S1 and S2 are 154.2 nm and 181.3 nm, respectively, while, the D(90) values and average size of S4 are up to 788.6 nm and 544.6 nm, respectively. Nanocrystalline C-S-H seed is a nano-cluster structure composed of a nano-chip unit, and larger size aggregates will be formed as the concentration of nano-chip units increases, which increase the size of the nano C-S-H. It was observed that the nano-sized C-S-H increased to the micron level as the inorganic concentration continued to increase, and that resulted in slight precipitation in the suspension.

**Figure 3:**
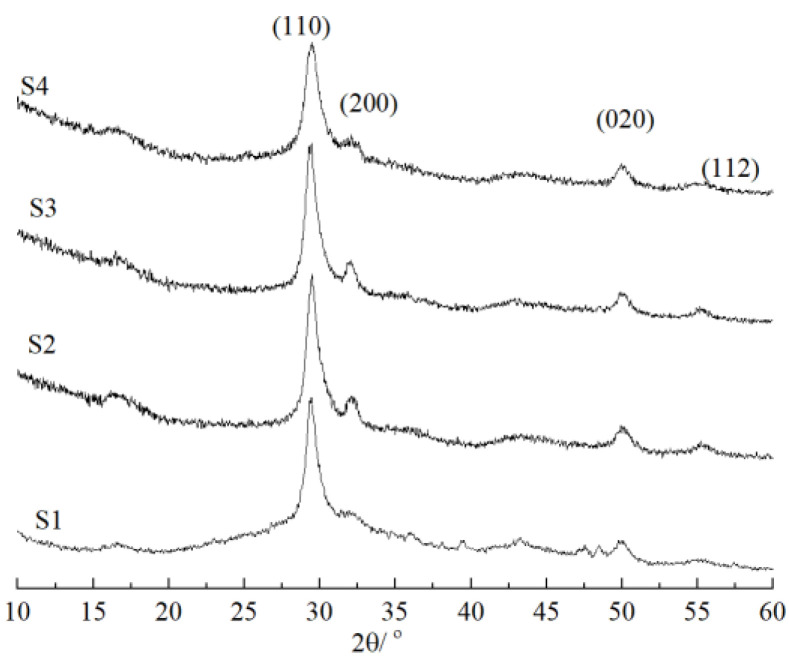
XRD patterns of nano-sized C-S-H.

The XRD spectra of nano-sized C-S-H are depicted in Figure 3, and the results presented here indicate that the synthetic C-S-H was similar to the structure of Tobermorite. The characteristic peaks in the XRD spectra are 29°, 32°, 49.7° and 55°, which correspond to the (110), (200), (020) and (112) planes, respectively [35], while only the characteristic diffraction peaks at 29° are obvious sharp peaks. Therefore, the XRD spectra of synthetic nano-sized C-S-H was semi-crystalline, which means it was in between the crystal structure and amorphous phases.

**Figure 4:**
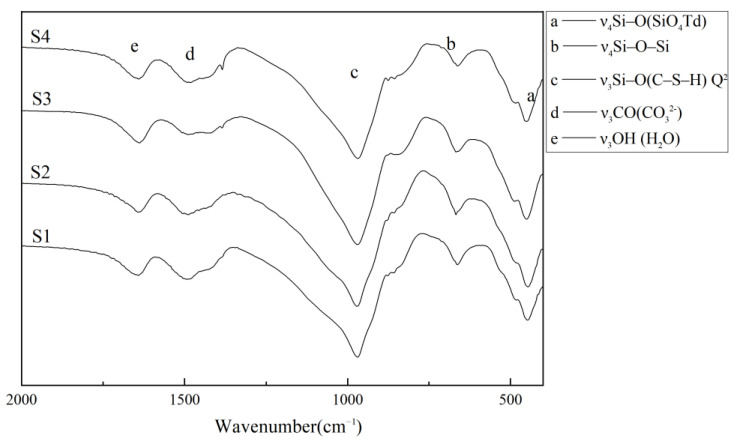
FT-IR spectrum of nano-sized C-S-H.

The FT-IR spectrum and the vibrations of synthetic C-S-H are depicted in Figure 4 and Table 4, respectively. There was no obvious difference in the the FT-IR spectrum, as shown in Figure 4. All the nano C-S-H specimens with different theoretical concentrations show absorption bands caused by bending vibration of Q^2^ at about 970 cm^−1^. Meanwhile, the out-of-plane bending vibration absorption bands of Si-O and Si-O-Si at 448 cm^−1^ and 670 cm^−1^ were observed, which indicated that C-S-H with high purity was synthesized. Slight carbonation reactions occurred due to the exposure to CO_2_; Therefore, weak absorption bands of CO_3_^2-^ vibration can be seen in the FT-IR analysis.

### 3.2. Hydration Process of Cement

**Figure 5:**
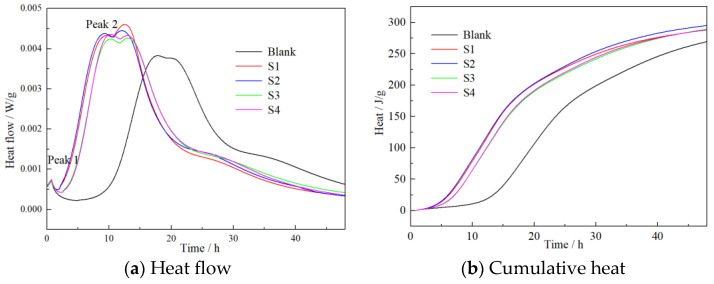
Effects of nano-sized C–S–H on the hydration heat of cement.

Characteristics of hydration heat flow and cumulative heat of reference paste and paste containing 5% S1, S2, S3 and S4 were tested at the same w/c ratio, and the results are depicted in Figure 5. The induction period of cement was significantly shortened and the second exothermic peak shifted to the left when nano C-S-H suspension was added. The second exothermic peak was mainly attributed to hydration of C_3_S, where C-S-H and Ca(OH)_2_ formed during the hydration process [36]. The heat flow indicated that nano-sized C-S-H accelerates the reaction of C_3_S effectively, especially within the 24 h of mixing. Accordingly, the 24 h cumulative heat of the paste containing S2 C-S-H was 44.6% higher than that of the reference paste.

### 3.3. Early Strength Enhancement of Nano C-S-H

**Figure 6:**
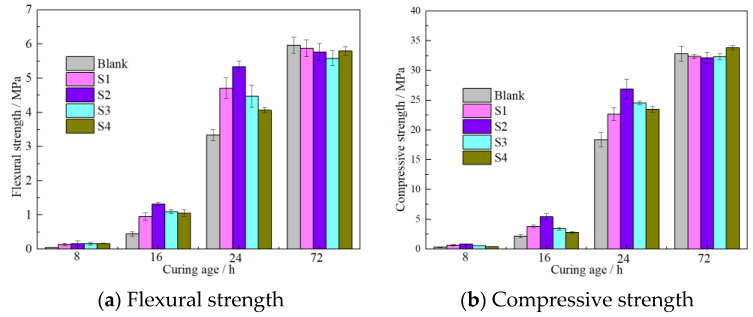
Effects of n-C-S-H on early mechanical properties of mortar.

The mechanical properties of cement mortar at early stages were examined, as depicted in Figure 6. Nano-sized C-S-H shows obvious enhancement in accelerating the hydration degree of cement at early periods, especially in the first 24 h. The enhancement effects decreased with increasing curing age. The 8 h, 16 h, 24 h and 72 h compressive strengths of mortars containing S2 increased by 176.0%, 145.6%, 43.9% and 13.0%, respectively, and this is consistent with the existing literature [37]. The enhancement by S1 is relatively weak compared with that of S2, due to its relatively lower theoretical C-S-H fraction. Although the average size of S2 is relatively larger, the enhancement effects are more obvious, which was caused by the high concentration of the effective constituent (C-S-H seed) in the S2 suspension. The enhancements by S3 and S4 were also relatively weak compared with that of S2, due to the larger particle sizes and weaker nucleation effects.

**Figure 7:**
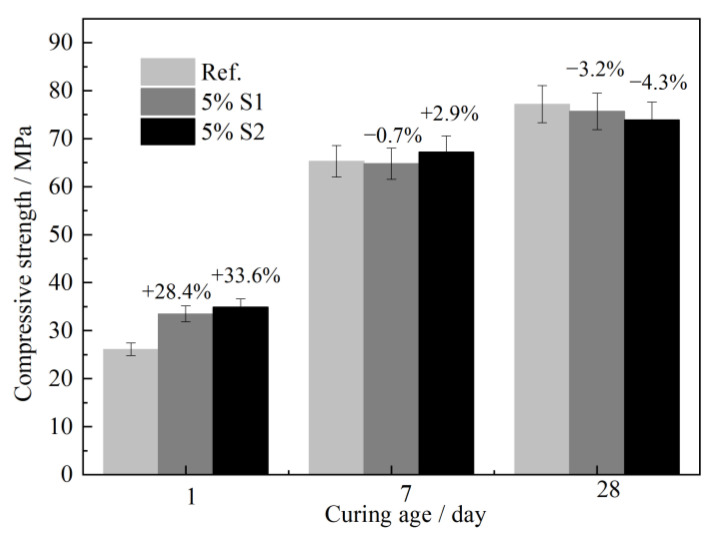
Effects of n-C-S-H on mechanical properties of concrete.

The concrete containing 5% S1 and S2 C-S-H suspensions were cured for 1 d, 7 d and 28 d; thereafter, the compressive strength were tested, as depicted in Figure 7. The results also confirmed that nano C-S-H can effectively increase the compressive strength of concrete at an early age (1 d). The 1 d compressive strength of concrete containing 50% S1 and S2 suspensions increased by 28.4% and 33.6%, respectively, compared to the reference concrete. Nevertheless, it showed slightly adverse effects on the 3 d and 28 d compressive strength. A decrease of −4.3% was observed for the 28 d compressive strength for concrete containing S2 C-S-H.

**Figure 8:**
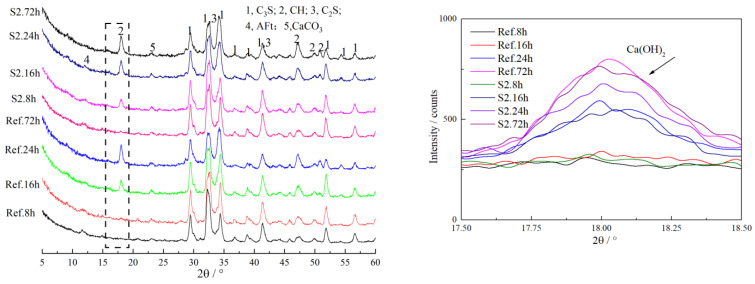
XRD patterns of cement paste.

The influence of nano C-S-H on the mineral compositions of hydrated pastes was explored, and the XRD patterns of pastes cured to for different ages are depicted in Figure 8. Results indicated that there were no obvious differences in the position and the shape of the characteristic diffraction peaks, indicating that there were no obvious effects on the hydration products. Meanwhile, the intensity of the characteristic diffraction peak of Ca(OH)_2_ was improved by nano-sized C-S-H significantly, in the first 1 d. The characteristic diffraction peak (2θ = 18°) of Ca(OH)_2_ in specimens containing S2 was obviously stronger than that of the reference paste, and the characteristic diffraction peaks (2θ = 32.5°) of the unhydrated C_3_S and C_2_S were much weaker than those of the reference samples. The XRD patterns showed that nano-sized C-S-H increased the hydration degree of cement primarily by accelerating the hydration degree of C_3_S, and the obvious increase in the compressive strength of concrete and mortars was also attributed to the higher hydration degree of C_3_S.

**Figure 9:**
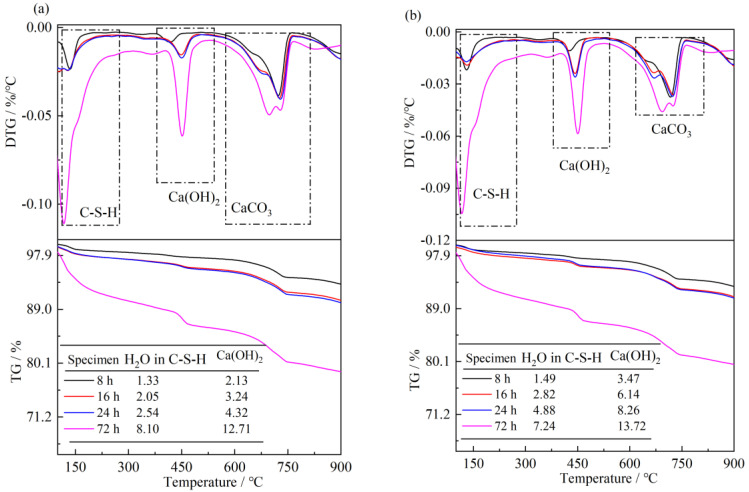
TG/DTG analysis of cement paste hydration for different periods: (**a**), reference paste; (**b**), paste containing S2 C-S-H).

Reference paste and paste containing nano-sized C-S-H(S2) samples cured to different ages were used for TG analysis. As shown in Figure 9a, the mass losses are mainly centered in the 100–280 °C, 400–500 °C and 580–800 °C ranges, and they were referred to the dehydration or decarbonation of amorphous C-S-H gel, Ca(OH)_2_ and CaCO_3_, respectively. Dehydration of C-S-H gel beyond 300 °C may also be possible; nevertheless, its content is much lower than CaCO_3_ as to be negligible [38], so we referred the mass losses in the 580–800 °C range to the decomposition of CaCO_3_. The fractions of the hydration products are always positively correlated to the hydration degree of the cement; therefore, a higher C-S-H gel and Ca(OH)_2_ content always shows a higher hydration degree [39]. The contents of the hydration products the in reference paste gradually increased with an increase in the hydration period. The 8 h, 16 h, 24 h, and 3 d Ca(OH)_2_ contents were 2.13%, 3.24%, 4.32% and 12.71%, respectively. Nano C-S-H suspension promoted the hydration reaction of paste during 8 h—24 h period. The 8 h, 16 h, 24 h Ca(OH)_2_ contents in pastes increased by 62.9%, 89.5% and 91.2%. The precise fractions of C-S-H gel in the hydration products cannot be calculated by the mass loss from TG data due to the ambiguous chemical formula. Therefore, the H_2_O fractions in the C-S-H structure, including interlayer water and crystalline water, were calculated and listed in Figure 8. The results indicated that the 8 h, 16 hand 24 h H_2_O fractions in paste increased by 12.6%, 37.3% and 53.1%, respectively, compared to the reference paste. No significant increase in Ca(OH)_2_ content was observed after hydration for 3 d, and it was consistent with the mechanical properties of mortar.

**Figure 10:**
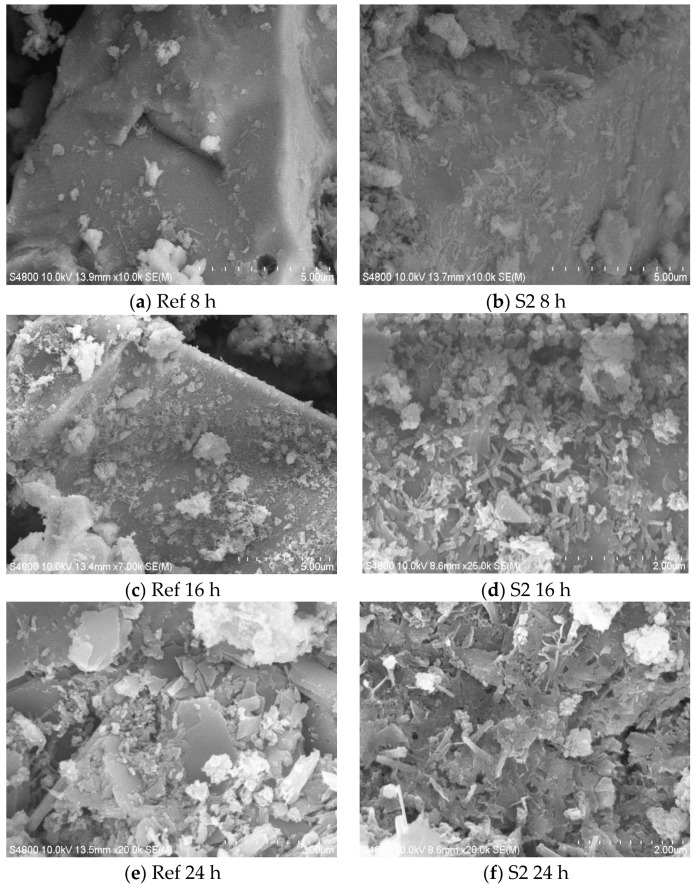

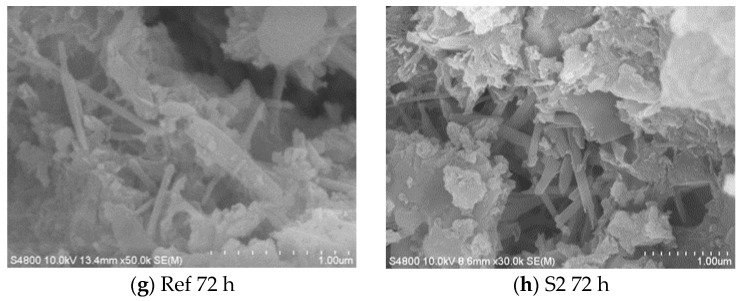
SEM patterns of cement paste at different ages.

Cement paste with/without S2 C-S-H suspensions were cured at 20 °C to specific ages, and then a fresh broken surface was sampled and the morphology of the hydrated cement paste was observed. The content of hydration products such as C-S-H gel, Ca(OH)_2_ and AFt are always positively correlated with its hydration degree. As shown in Figure 10a,b, unhydrated cement clinker and very small amounts of hydration products could be clearly seen after 8 h of hydration. Nevertheless, the amount of hydration products in paste containing S2 was more than that in reference paste, confirming that the hydration processes before 8 h were promoted by nano-sized C-S-H. A similar phenomenon was observed in paste hydrated for 16 h (Figure 10c,d). The total amount of unhydrated cement particles decreases obviously and the matrix was denser than that of the reference paste. Therefore, the nano-sized C-S-H can effectively accelerate the hydration reaction and generate more hydration products to fill the voids, thus increasing the early compressive strength of mortar and concrete.

The morphology of paste after curing for 72 h indicated that needlelike Ca(OH)_2_ and flocculent C-S-H gel were the dominant hydration products in the reference paste and paste containing S2 C-S-H. The most obvious difference is that AFt is separated with the amorphous C-S-H gel in the reference paste, and many large pores could be observed in the hardened paste. Nevertheless, in the paste containing S2 C-S-H suspension, as is show in Figure 10h, C-S-H fills in the pore structure, making the pores in the matrix smaller and the structure of the paste denser.

### 3.4. Early Strength Enhancement of Nano-Sized C-S-H at 10 °C

**Figure 11:**
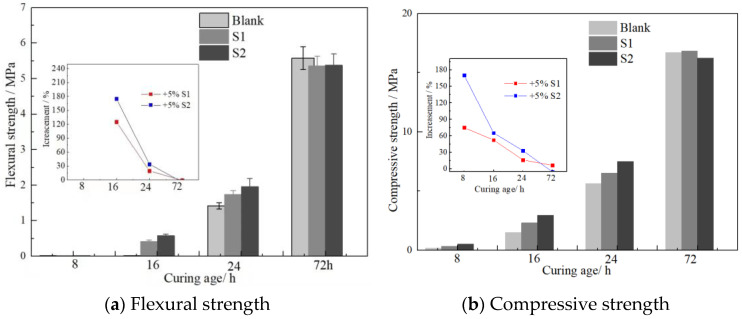
Effects of n-C-S-H on mechanical properties of mortar cured at 10 °C.

The compressive strength of mortars cured at 10 °C were tested. Average values of mechanical properties were reported, and the results are presented in Figure 11. Nano C-S-H can effectively increase the compressive strength and flexural strength of mortars to varying degrees in the first 24 h. The 8 h, 16, 24 h and 72 h compressive strength of mortars containing S2 increased by 170.0%, 65.4%, 33.2% and −5.2%, respectively. However, the enhancement effect of nano C-S-H for mortars cured at 10 °C was much lower than that for mortars cured at 20 °C.

**Figure 12:**
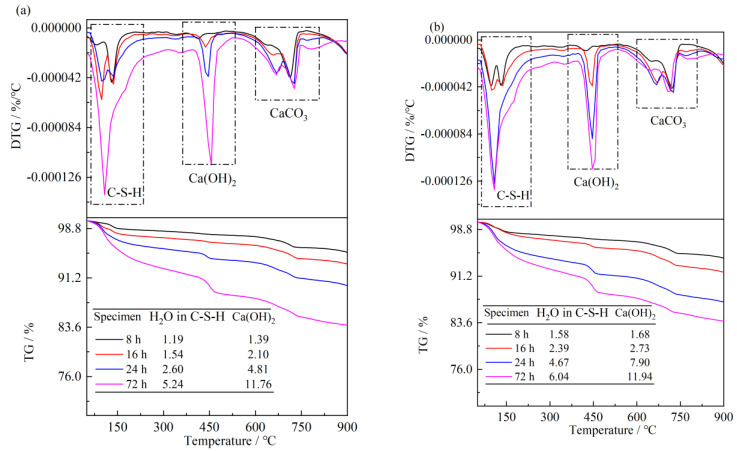
TG/DTG curves of cement paste hydration at 10 °C: (**a**), reference paste; (**b**), paste containing nano S2 C-S-H.

To further confirm the abovementioned conclusions, TG analyses were performed to calculate the content of Ca(OH)_2_ and C-S-H gel, which are the dominant hydration products and reflect the hydration degree of cement. The TG curves (50–1000 °C) are depicted in Figure 12. The mass losses were similar to those of the paste cured at 20 °C. The TG data indicated the 8 h, 16 h, 24 h and 3 d Ca(OH)_2_ contents in the reference hydrated paste were 1.39%, 2.10%, 4.81% and 11.76%, respectively, lower than those of the reference paste cured at 20 °C. Meanwhile, the 8 h, 16 h and 24 h Ca(OH)_2_ contents in pastes containing 5% S2 C-S-H suspension increased by 20.9%, 30.0% and 64.2%, respectively, compared to the reference paste. the TG/DTG data were consistent with the variations in the compressive strength of mortars. Therefore, nano C-S-H can also promote hydration at 10 °C; however, the enhancement effect is weaker than that at 20 °C.

## 4. Conclusions

Nano-sized C-S-H suspensions with different fractions and size distributions were prepared and the structure was tested by zeta potential, XRD and FT-IR. The effects of nano-sized C-S-H on the exothermic hydration heat of cement, early strength development and hydration products of mortars at different temperatures were studied. The main conclusions of our work are listed below:

1. Nano-sized C-S-H with semi-crystalline structures were prepared. The average size of nano-sized C-S-H increased with an increase in the theoretical concentration, and a slight precipitation in the suspension was observed when the theoretical concentration was 2%. The D(90) values of S1 and S2 are 105.2 nm and 117.3 nm, respectively, and the average sizes of S1 and S2 are 154.2 nm and 181.3 nm, respectively.
2. Nano-sized C-S-H promotes the hydration process of cement mainly by accelerating the reaction degree of C_3_S, especially within the first 24 h of casting. The 8 h, 16 h, 24 h Ca(OH)_2_ contents in hydrated cement increased by 62.9%, 89.5% and 91.2%, respectively.
3. The 8 h, 16 h and 24 h compressive strength of mortars containing nano-sized C-S-H increased by 176.0%, 145.6% and 43.9%, respectively, compared with the reference mortar, while the 3 d flexural strength and compressive strength show a slightly decrease.
4. Nano-sized C-S-H improved the hydration degree of Portland cement cured at 10 °C, but the enhancement was much lower than for that cured at 20 °C.

## Figures and Tables

**Table 1:** Chemical compositions of Portland cements (wt %).

SiO_2_	CaO	MgO	Fe_2_O_3_	Al_2_O_3_	K_2_O	Na_2_O	SO_3_	LOI
20.31	62.58	2.93	3.73	4.38	-	-	2.45	1.58

**Table 2:** Chemical compositions of grade I fly ash and ultrafine slag (wt %).

Specimen	Na_2_O	MgO	Al_2_O_3_	SiO_2_	P_2_O_5_	K_2_O	CaO	Fe_2_O_3_
Grade IFly ash	1.75	1.46	26.54	48.92	0.15	2.03	4.82	4.59
Ultrafine slag	-	8.43	1.24	32.21	-	-	37.52	15.28

**Table 3:** Mix ratio of concrete (kg/m^3^).

Mix Design	Cement	Fly ash	Slag	Gravel	Sand	Water	AEa	n-C-S-H^b^
H0	384	72	24	1020	724	136	2.4	0
H1	384	72	24	1020	724	136	2.4	24, S1
H2	384	72	24	1020	724	136	2.4	24, S2

AEa, The type of water reducer; n-C-S-H^b^, n-C-S-H suspension.

**Table 4:** FT-IR spectra and assignments of nano C-S-H (cm^−1^).

Band	Specimen				Assigned to
	S1	S2	S3	S4	
a	449	452	451	451	ν_4_Si–O (SiO_4_Td)
b	671	671	671	670	ν_4_Si–O–Si
c	968	970	972	966	ν_3_Si–O(C–S–H) Q^2^
d	1489	1471	1426	1427	ν_3_CO (CO_3_^2-^)
e	1641	1640	1639	1640	ν_3_OH (H_2_O)

## Data Availability

Not applicable.
